# Urban Stress Indirectly Influences Psychological Symptoms through Its Association with Distress Tolerance and Perceived Social Support among Adults Experiencing Homelessness

**DOI:** 10.3390/ijerph17155301

**Published:** 2020-07-23

**Authors:** Daphne C. Hernandez, Sajeevika S. Daundasekara, Michael J. Zvolensky, Lorraine R. Reitzel, Diane Santa Maria, Adam C. Alexander, Darla E. Kendzor, Michael S. Businelle

**Affiliations:** 1 Cizik School of Nursing, The University of Texas Health Science Center, Houston, TX 77030, USA; diane.m.santamaria@uth.tmc.edu; 2 Department of Health & Human Performance, University of Houston, Houston, TX 77204, USA; ssdaunda@central.uh.edu; 3 Department of Psychology, University of Houston, Houston, TX 77204, USA; mjzvolen@central.uh.edu; 4 HEALTH Research Institute, University of Houston, Houston, TX 77204, USA; lrreitze@central.uh.edu; 5 Department of Psychological, Health, and Learning Sciences, University of Houston, Houston, TX 77204, USA; 6 Oklahoma Tobacco Research Center, Stephenson Cancer Center, University of Oklahoma Health Sciences Center, Oklahoma, OK 73104, USA; Adam-Alexander@ouhsc.edu (A.C.A.); Darla-Kendzor@ouhsc.edu (D.E.K.); Michael-Businelle@ouhsc.edu (M.S.B.)

**Keywords:** urban life stress, perceived social support, major depressive disorder, distress, PTSD, structural equation modeling, buffering hypothesis, intrapersonal skills, interpersonal skills, homeless

## Abstract

Traditionally, intrapersonal characteristics (distress tolerance) and interpersonal characteristics (social support) have been studied separately rather than simultaneously. In the current study, we address this gap by simultaneously examining these characteristics as potential indirect associations linking established urban stress–depression and urban stress–Post-Traumatic Stress Disorder (PTSD) relationships. Adults experiencing homelessness were recruited from six homeless shelters in Oklahoma City (n = 567). Participants self-reported urban life stress (Urban Life Stress Scale), distress tolerance (Distress Tolerance Scale), social support (Interpersonal Support Evaluation List 12), major depressive disorder (Patient Health Questionnaire-8), and PTSD symptoms (Primary Care Post-Traumatic Stress Disorder screener). Covariate-adjusted structural equation models indicated a significant indirect effect of distress tolerance on the urban stress–depression (b = 0.101, 95% CI = 0.061, 0.147) and urban stress–PTSD (b = 0.065, 95% CI = 0.023, 0.112) relationships. Additionally, a significant indirect effect of social support on the urban stress–depression (b = 0.091, 95% CI = 0.053, 0.133) and urban stress-PTSD relationships (b = 0.043, 95% CI = 0.006, 0.082) was evident. Further, both the urban stress–depression (b = 0.022, 95% CI = 0.011, 0.037) and urban stress–PTSD relationships (b = 0.014, 95% CI = 0.005, 0.026) were associated indirectly through social support to distress tolerance. Interventions that aim to increase social support may also increase distress tolerance skills and indirectly reduce depressive and PTSD symptoms in the context of urban stress among adults experiencing homelessness.

## 1. Introduction

According to the 2018 point-in-time estimates, 552,830 people experienced homelessness in the United States [1]. Compared to domiciled adults, those experiencing homelessness are disproportionately affected by poor mental and physical health [2,3,4,5]. According to previous research, 48–65% of adults experiencing homelessness reported diagnoses of depression, and 43% reported diagnoses of Post-Traumatic Stress Disorder (PTSD) [2,3,6]. Individuals experiencing homelessness are at risk of mental distress due to challenges associated with meeting their daily needs, including food, shelter, and health care, and stressors related to living on the streets or in shelters [7].

Research evidence indicates that urbanization may lead to mental health problems, including psychotic experiences, depression, and stress-related disorders, especially among vulnerable individuals [8]. In general, individuals living in urban settings frequently experience environmental and social stressors that could negatively affect their mental health. The tension that results from living in an urban environment is referred to as urban stress. Urban life-related stressors include, but are not limited to, the poor-quality built environment, pollution, violence, substance use, racism, and discrimination. Studies have shown that chronic exposure to such community-level urban stressors may lead to depression among domiciled and homeless populations [9,10,11,12,13]. However, evidence is scarce on the possible links underlying the association between urban life stress and mental health problems. Thus, the purpose of this study was to evaluate two potential factors (distress tolerance and social support) that may serve as links underlying the association between urban life stress and depression/PTSD among adults experiencing homelessness.

A transdiagnostic individual difference construct that makes an individual more susceptible to stress and a negative mood is distress tolerance. Distress tolerance refers to the perceived or actual ability to withstand negative emotions and other aversive stimuli [14]. Lower perceived distress tolerance has been associated with various psychological symptoms, including depression and PTSD [15,16,17,18]. In the limited available research, lower perceived distress tolerance has been identified as a link between sleep problems and stress [19]. Further, distress tolerance has shown significant indirect effects on the association between food insecurity and physical and mental health among adults who experience homelessness [20]. Despite the relevance of urban stress to adults who are homeless, research on the association between urban stress and distress tolerance is lacking. Further, past work has not examined whether lower distress tolerance has a significant indirect effect on the association between (1) urban stress and depression and (2) urban stress and PTSD.

Evidence indicates that perceived social support can attenuate negative psychological outcomes related to undesirable life events [21]. Individuals may receive social support in the form of structural support or functional support [22,23]. Although structural support focuses on the number and frequency of contacts with social networks, functional support focuses on the perceived function that the social networks serve in providing support. Functional support includes appraisal (i.e., the perceived availability of advice and guidance), belonging (i.e., empathy, acceptance, concern from others), and tangible support (i.e., the perceived availability of material aid) [22,23]. Functional support has been demonstrated to have a significant positive effect on self-reported health status, preventing the onset of cardiovascular disease, and is associated with lower diabetes prevalence [24,25,26]. In comparison to domiciled adults, those experiencing homelessness are often socially isolated and perceive less social support [27,28]. Research among individuals experiencing homelessness has shown that social support is associated with better physical and mental health and a lower likelihood of victimization [29,30]. Further, several studies have demonstrated that social support attenuates the negative relationship between various types of stressors and psychological/physiological outcomes, or what is referred to as the stress-buffering hypothesis [31,32,33]. However, no studies have assessed whether social support attenuates the negative relationships between urban stress and depression, and urban stress and PTSD.

Distress tolerance can be considered an intrapersonal characteristic and social support an interpersonal characteristic; thus, there is a tendency to study them separately [34]. Few studies have looked at these two characteristics simultaneously. For instance, a research study on adolescents following a natural disaster found that lower distress tolerance and lower social support were related to elevated depression symptoms [35]. Among a sample of older adults, social support had a direct effect on distress tolerance and mental health [36]. Research has also demonstrated that the absence of a support network could further compound the adverse health outcomes associated with stressors among women experiencing homelessness [37]. An individual with lower distress tolerance may not have the intrapersonal ability to handle additional stressors nor adequate social support to stave off depression. Further, social support could influence distress tolerance, whereby inadequate social support could directly negatively influence distress tolerance and contribute to psychological symptoms when stressors arise. However, models have not evaluated the indirect association of social support and distress tolerance simultaneously on urban stress and psychological symptoms among adults experiencing homelessness. Research is also lacking on whether low social support directly lowers distress tolerance, thereby placing vulnerable populations at further risk of experiencing depression and PTSD when presented with urban stress.

The current study contributes to the literature by simultaneously examining an intrapersonal characteristic (distress tolerance) and an interpersonal characteristic (social support) for potential associations between urban stress and depression and between urban stress and PTSD among a sample of adults experiencing homelessness. Building on prior studies [15,16,17,18,21,29,30,36], the current study was designed to evaluate the following hypotheses:

1. Greater urban stress would be associated with increased likelihood of psychological symptoms (i.e., depression and PTSD).
2. Greater urban stress would be associated with lower distress tolerance, and greater distress tolerance would be associated with decreased likelihood of psychological symptoms. Thus, the negative impact of urban stress on psychological symptoms would be associated through lower distress tolerance.
3. Greater urban stress would be associated with lower perceived social support, and greater perceived social support would be associated with decreased likelihood of psychological symptoms. Thus, the negative impact of urban stress on psychological symptoms would be associated via lower social support.
4. Lower perceived social support would be associated with lower distress tolerance. The negative impact of urban stress on psychological symptoms would be associated through the impact of social support on distress tolerance.

## 2. Materials and Methods

### 2.1. Study Participants and Data Collection

Participants were recruited from six homeless shelters in Oklahoma City, Oklahoma, from July 2016 until August 2016. Research staff met interested participants at the shelters and screened for eligibility. Individuals were eligible to participate in the study if they: (a) were 18 years or older, (b) were receiving services at targeted homeless shelters, and (c) had a 7th grade or higher English literacy level, which was defined as a score ≥4 on the Rapid Estimate of Adult Literacy in Medicine-Short Form (REALM-SF) [38,39]. Research staff provided additional study details to potential participants and completed the informed consent procedure. Eligible participants completed the study questionnaire via a laptop or tablet, wore headphones, and listened to the program read each survey item as it appeared on the screen. The screening and survey took about an hour to complete, and each participant was compensated with a USD 20 department store gift card. The Institutional Review Boards at the University of Oklahoma Health Sciences Center (IRB#6939) and the University of Houston (IRB#8454-16499-01) approved the study.

Overall, 648 individuals were screened for the study, and 38 were excluded due to lower literacy levels. Among the 610 participants that met the eligibility criteria, 29 were excluded as they were not currently homeless (defined as an individual who does not have a personal residence or other permanent location to sleep), and 14 were excluded due to missing data. The analyzable sample consisted of 567 adults with complete data who were currently homeless and receiving services at one of the targeted homeless shelters.

### 2.2. Measures

#### 2.2.1. Major Depressive Disorder

Major depressive disorder was assessed using the eight-item Patient Health Questionnaire (PHQ) [40]. Those who scored 10 points or higher (score range = 0–24) were categorized as having probable major depression [41].

#### 2.2.2. PTSD Symptoms

Symptoms of PTSD were assessed using the four-item Primary Care Post-Traumatic Stress Disorder (PC-PTSD) screener. The response options for each item were “yes” and “no.” Those who responded “yes” to three or more items (score range = 0–4) were categorized as experiencing PTSD symptoms [42].

#### 2.2.3. Urban Life Stress

Urban life stress was measured using the Urban Life Stress Scale (ULSS) [43]. The scale measures subjective contextual community-level stressors as potential sources of chronic stress experienced by persons living in medium to large cities. Sample items include, “In your day to day life, how much stress do you generally experience related to money or finances?” and “In your day to day life, how much stress do you generally experience related to your housing or living situation?” Responses were recorded using a 5-point scale ranging from 1 = no stress at all to 5 = extremely stressful–more than I can handle, with higher scores indicating a greater level of stress associated with living in an urban environment (possible score range = 1–105).

#### 2.2.4. Distress Tolerance

Participants’ emotional distress tolerance was assessed using the 16-item self-reported Distress Tolerance Scale. The scale defines distress tolerance as one’s ability to withstand emotional distress on a 5-point scale ranging from 1 = strongly disagree to 5 = strongly agree. Sample items include, “Feeling distressed or upset is unbearable to me,” and “When I feel distressed or upset, all I can think about is how bad I feel.” The negatively stated items were reverse-scored to obtain the average total score (possible range = 1–5), with higher scores indicating higher levels of distress tolerance [44].

#### 2.2.5. Perceived Social Support

The Interpersonal Support Evaluation List (ISEL)-12 was used to measure the perceived availability of social support. The scale consists of 12 items and 3 subscales: 1. the tangible support subscale (e.g., “If I were sick, I could easily find someone to help me with my daily chores“), 2. the belonging subscale (e.g., “If I wanted to go on a trip for a day, for example, to the country or mountains, I would have a hard time finding someone to go with me”), and 3. the appraisal subscale (e.g., “I feel that there is no one I can share my most private worries and fears with”). Items were rated on a four-point scale (1 = definitely false to 4 = definitely true), and the negatively stated items were reverse-scored. The total score ranges from 12 to 48, and higher scores indicate greater social support [45].

#### 2.2.6. Covariates

Variables that may affect health outcomes, urban stress, distress tolerance, or social support were included as covariates in the models. Several studies have shown that socio-demographic characteristics of adults experiencing homelessness are associated with their mental health diagnosis, distress tolerance, and social support [19,46,47]. The socio-demographic covariates included were: age (in years), sex (1 = female, 0 = male), race/ethnicity (1 = white/non-minority, 0 = minority race/ethnicity), marital status (1 = married, 0 = not married), level of education (1 = less than high school, 0 = high school or higher), employment (1 = unemployed/disability limits employment, 0 = employed), and source of income (1 = no source of income, 0 = at least one source of income). In addition, models controlled for the effect of the total amount of time spent homeless (in years), health insurance (1 = uninsured, 0 = insured to any extent), shelter services utilization (number of services used), alcohol abuse/dependence, and smoking. These variables have also been associated with mental health symptoms, distress tolerance, and social support among homeless individuals [19,48,49,50]. Alcohol abuse/dependence was determined based on seven items from the PHQ [40]. Participants with two or more affirmative answers were categorized as having probable alcohol abuse or dependence [40]. Two questionnaire items were used to determine the current smoking status: “Have you smoked at least 100 cigarettes (or cigarillos) in your entire life?” and “Have you smoked cigarettes or cigarillos in the past 30 days?” Those who responded affirmatively to both questions were categorized as current smokers.

### 2.3. Statistical Analysis

The sample descriptive statistics and zero-order correlations among study variables were examined using SPSS version 26.0 (IBM Corp, Armonk, NY, USA) [51]. Inter-correlations between main study variables and covariates were evaluated using Pearson’s correlation for associations between continuous variables, point-biserial correlations for continuous-binary associations, and Phi/Cramer V coefficients for associations between binary variables.

**Figure 1:**
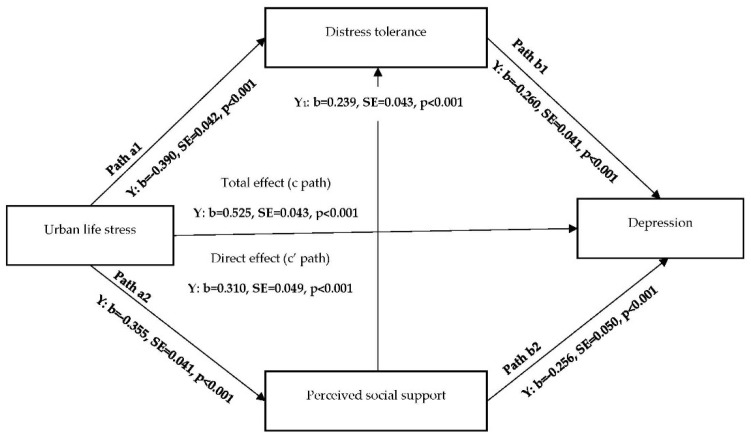
Covariate-adjusted structural equation model predicting depressive symptoms through distress tolerance and perceived social support. Note: b = Standardized Coefficient, SE = Standard Error. Higher scores indicate greater urban life stress, distress tolerance and perceived social support.

**Figure 2:**
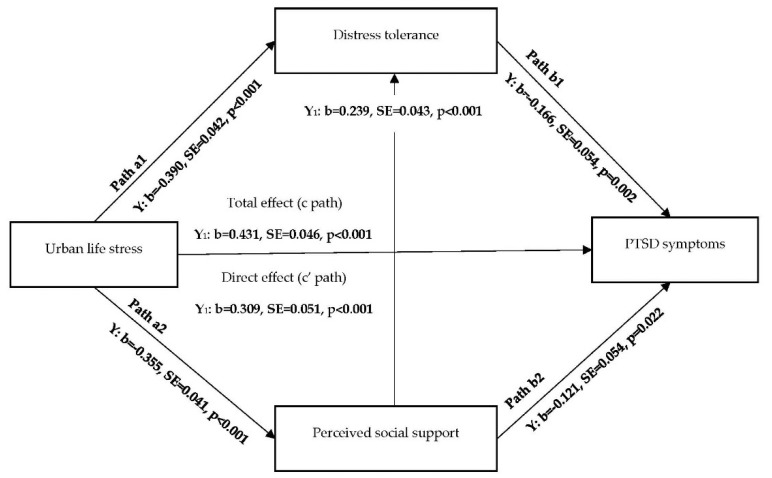
Covariate adjusted structural equation model predicting PTSD symptoms through distress tolerance and perceived social support. Note: b = Standardized Coefficient, SE = Standard Error, PTSD = Post-Traumatic Stress Disorder. Higher scores indicate greater urban life stress, distress tolerance and perceived social support.

Two structural equation models were used to test the indirect effects of urban life stress on major depressive disorder and PTSD symptoms via distress tolerance and perceived social support (Figure 1 and Figure 2). Initially, models were evaluated without including the covariates (unadjusted models), followed by covariate-adjusted models. The aforementioned covariates (i.e., age, sex, race/ethnicity, marital status, level of education, employment, source of income, total years being homeless, health insurance, shelter service use, alcohol abuse, and smoking) were included in all pathways. Maximum likelihood estimation with bootstrapping (using 5000 resamples of data) was used in the indirect effect analyses to account for possible non-normality of the effect distributions due to binary outcome variables [52]. The models were evaluated using Mplus version 8.2 (Muthén & Muthén, Los Angeles, CA, USA) [53].

Standardized path coefficients for the effect of the predictor on mediators (a path), the effect of mediators on the outcome controlling for the predictor (b path), the effect of the predictor on the outcome without controlling for the mediators (path c—total effect), and the effect of the predictor on the outcome controlling for the mediators (path c—direct effect) were determined for each health outcome (Figure 1 and Figure 2). A significant indirect effect (path a × b) was evidenced by a 95% bootstrap Confidence Interval (CI), not containing zero [54]. The specific indirect effects of perceived social support and distress tolerance were compared using the Model Constraint command in Mplus. The specified models contained the maximum number of possible pathways (“just-identified” models). Thus, we were unable to assess model fit indices.

## 3. Results

### 3.1. Descriptive Analysis

The study sample characteristics are shown in Table 1. Most of the participants were men (n = 360, 64%), white/non-minority (n = 321, 57%), not married (n = 497, 88%), and unemployed (n = 501, 88%). Almost a third (n = 174, 31%) of the adults experiencing homelessness had symptoms consistent with probable major depression, and 33% (n = 184) screened positive for PTSD. The mean distress tolerance level was 3.10 (Standard Deviation (SD) = 0.98), and the mean perceived social support score was 32.95 (SD = 8.73).

Table 2 presents the inter-correlations between participant characteristics. There were significant moderate, positive correlations between the health outcome variables. The point-biserial correlation coefficient indicates a moderate, positive correlation between urban life stress and depression. There was a small positive correlation between urban life stress and PTSD symptoms. Both distress tolerance and perceived social support had significant small negative correlations with the depression and PTSD symptoms (Table 2).

### 3.2. Indirect Effect Model for Major Depressive Disorder 

According to the unadjusted model, there was a significant total effect (b = 0.522, *p* < 0.001) and direct effect (b = 0.303, *p* < 0.001) of urban stress on probable major depressive disorder. All the a and b paths of the model and the indirect effects through distress tolerance and social support were also statistically significant (*p* < 0.05; results not shown). Therefore, the model was further evaluated using the covariate-adjusted model. Results did not differ substantively in unadjusted and adjusted models. Thus, the adjusted model results are illustrated in Figure 1 and Table 3.

According to the covariate-adjusted model, greater urban life stress was significantly associated with greater likelihood of probable major depressive disorder (total effect; b = 0.525, *p* < 0.001). The association continued to be significant after adjusting for distress tolerance and perceived social support, in addition to the covariates (direct effect; b = 0.310, *p* < 0.001) (Figure 1). Greater urban life stress was also associated with lower distress tolerance (path a1; b = −0.390, *p* < 0.001) and perceived social support (path a2; b = −0.355, *p* < 0.001). Greater distress tolerance and greater perceived social support were associated with lower likelihood of probable major depressive disorder (path b1; b = −0.260, *p* < 0.001 and path b2; b = −0.256, *p* < 0.001). Perceived social support was positively associated with distress tolerance (b = 0.239, *p* < 0.001), indicating that greater social support was associated with greater distress tolerance.

Urban life stress indirectly influenced major depressive disorder through distress tolerance controlling for perceived social support (b = 0.101, 95% CI = 0.061, 0.147) and through perceived social support controlling for distress tolerance (b = 0.091, 95% CI = 0.053, 0.133) (Table 3). There was also a significant indirect association between urban life stress and major depressive disorder through the concurrent association between perceived social support and distress tolerance (b = 0.022, 95% CI = 0.011, 0.037). Distress tolerance accounted for 33.1% of the total indirect effects and 11.6% of the total effects of urban life stress on major depressive disorder. Perceived social support accounted for 30.3% of the total indirect effects and 10.6% of the total effects of urban life stress on major depressive disorder. There was no significant difference in the indirect effect of distress tolerance and perceived social support (b = −0.002, *p* = 0.771).

### 3.3. Indirect Effect Model for PTSD Symptoms

According to the unadjusted model, there was a significant total effect (b = 0.452, *p* < 0.001) and direct effect (b = 0.332, *p* < 0.001) of urban stress on PTSD symptoms. All the a and b paths of the model and the indirect effects through both distress tolerance and social support were also statistically significant (*p* < 0.05; results not shown). Therefore, the model was further evaluated using the covariate-adjusted model. Results did not differ substantively in unadjusted and adjusted models. Thus, the adjusted model results are illustrated in Figure 2 and Table 4.

According to the covariate-adjusted model, greater urban life stress was significantly associated with greater likelihood of PTSD symptoms (path c; b = 0.431, *p* < 0.001). The association continued to be significant after adjusting for distress tolerance and perceived social support in addition to the covariates (path c’; b = 0.309, *p* < 0.001) (Figure 2). Greater urban life stress was associated with lower distress tolerance (path a1; b = −0.390, *p* < 0.001) and lower perceived social support (path a2; b = −0.355, *p* < 0.001). Greater distress tolerance and greater perceived social support were associated with lower likelihood of PTSD symptoms (path b1; b = −0.166, p = 0.002 and path b2; b = −0.121, *p* = 0.022). Perceived social support was positively associated with distress tolerance (b = 0.239, *p* < 0.001).

Urban life stress indirectly influenced PTSD symptoms through distress tolerance controlling for perceived social support (b = 0.065, 95% CI = 0.023, 0.112) and through perceived social support controlling for distress tolerance (b = 0.043, 95% CI = 0.006, 0.082) (Table 4). There was also a significant indirect association between urban life stress and PTSD symptoms through the concurrent association of perceived social support and distress tolerance (b = 0.014, 95% CI = 0.005, 0.026). Distress tolerance accounted for 53.3% of the total indirect effect and 15.1% of the total effect of urban life stress on PTSD. Perceived social support accounted for 35.2% of the total indirect effect and 10.0% of the total effect of urban life stress on PTSD. There was no significant difference in the indirect effect of distress tolerance and perceived social support (b = −0.003, *p* = 0.527).

## 4. Discussion

Individuals experiencing homelessness are among the most vulnerable populations in a society. They experience an elevated risk of psychological distress due to challenges associated with living on the streets or in shelters [7], including physical, sexual, or emotional abuse/maltreatment, violence, racism, drug/alcohol misuse, and discrimination [11,12,13,55]. As the majority of homeless adults live in urban environments, they also experience higher levels of stress associated with day-to-day life in urban environments and exposure to environmental adversities [56,57]. In addition, several research findings have indicated higher rates of psychopathology and lower levels of social support and ability to withstand distress in this population [58]. The characteristics of the current sample support these findings, specifically a higher rate of major depression and PTSD, greater urban life stress, and lower distress tolerance and social support among adults experiencing homelessness.

To our knowledge, this is the first study that simultaneously explored distress tolerance (intrapersonal characteristic) and social support (interpersonal characteristic) as potential associations underlying the urban stress-depression/PTSD relationship among adults experiencing homelessness. Similar to prior studies [31,32,33], social support buffered the relationship between urban stress and depression and urban stress and PTSD. Although adults experiencing homelessness generally do not have a large network of support that they can turn to compared to domiciled adults [30,59], those who have a reliable network that can provide functional support experience a lower risk of depression and PTSD symptoms in the presence of urban stress. On the other hand, lower distress tolerance exacerbated the relationship between urban stress and depression and urban stress and PTSD symptoms. However, the present study cannot rule out the potential bidirectionality of the associations between the main study variables. Longitudinal research is necessary to assess the potential causal relationships, and the use of sophisticated models (e.g., cross-lagged panel models) may prevent the over- or underestimation of effects. Irrespective of these limitations, the current study results provide preliminary evidence suggesting that adults who experience homelessness and have a lower ability to tolerate distress and/or lack social support may be at a higher risk of experiencing depression and PTSD when experiencing urban stressors. This finding is comparable to previous research that suggested lower distress tolerance may be an important indirect factor between a stressor and a health indicator [19,20] and expands it to urban stress and psychological symptomatology. Thus, homeless adults living in an urban setting might benefit from healthy coping strategies and emotional regulation skills. For example, cognitive behavioral approaches have shown promise when applied to regulating distress among vulnerable populations [15]. Providing adults who experience homelessness with mindfulness-based cognitive therapy [60] or another cognitive-based behavioral therapy could assist with regulating emotions. In addition, eye movement desensitization and reprocessing therapy has been shown to reduce subjective distress among homeless individuals and thus might assist with regulating emotions among this population [61].

The results of the current study also showed that while greater urban stress is related to less social support, social support is associated with greater distress tolerance, which then may be associated with lower likelihood of major depressive disorder and PTSD symptoms. It could be that the interpersonal skills used to build social support, such as communication techniques, contribute to informing intrapersonal skills through self-awareness and introspection. The inter-relatedness of the two skills contributes to greater emotional intelligence, resulting in better psychological adaptation [62]. Thus, increasing functional support could improve distress tolerance among adults experiencing homelessness, ultimately improving psychological symptoms.

Providing functional support, however, may be more difficult among adults who experience homelessness compared to domiciled adults. This is because social support may also have negative effects on adults who experience homelessness [37,63,64]. Specifically, studies have shown that social networks may operate differently for substance abusers [65]. Many individuals experiencing homelessness do not have a supportive individual or a support network [30,59]. Consequently, they may get support from individuals who are themselves at risk of poor social and emotional health and negative health behaviors, thereby placing them at higher risks of these maladaptive coping methods [66]. Among substance abusers, support from individuals who advocate ‘‘abstinence’’ or ‘‘responsible use” may promote positive outcomes; on the other hand, support from persons who are active substance users/abusers may lead to escalated substance use/abuse. Therefore, while social support can be beneficial, suggesting individuals or networks as support systems needs to be undertaken with caution.

The study had some limitations that need to be considered when interpreting these findings. The data for the current study were collected from adults experiencing homelessness in Oklahoma City, OK, and the sample was limited to those receiving services at the selected shelters. Therefore, the current results may not be generalizable to all adult homeless populations in the U.S. Further, data were collected through participants’ self-reported measures; thus, bias could have resulted from social desirability. However, all the measures used for data collection have been validated for accuracy and reliability. Due to the cross-sectional study design, it is not possible to determine the directionality of the relationships among study variables. Findings are suggestive and provide a starting point for further investigation. Longitudinal studies are required to understand the directionality of the associations among urban stress, social support, distress tolerance, and psychological symptoms in adults experiencing homelessness. Further, interventions designed to improve social support and/or distress tolerance would provide more insight into the interactions between these constructs.

### Implications for Practice

Adults experiencing homelessness residing in urban environments experience higher levels of stress associated with urban living. Shelters providing services to this population need to incorporate strategies to help these individuals cope with these specific types of stressors in addition to general stressors associated with street/shelter living. Most of the current interventions to improve the mental health status among homeless adults include providing housing, improving social support, and providing instrumental support as well as counselling [67]. Designing such interventions in the future may require targeting higher levels of stress associated with urban living and lower distress tolerance, in combination with providing housing and social support to better serve the mental health needs of this population.

## 5. Conclusions

The current study indicates that greater social support is associated with lower risk of psychological symptoms in the presence of urban stress, while lower distress tolerance is associated with higher risk of experiencing psychological symptoms when urban stress exists. Moreover, providing social support may positively influence the ability to tolerate stress associated with urban stressors, thereby reducing the risk of experiencing depression and PTSD symptoms among adults experiencing homelessness. Thus, interventions targeting urban homeless adults with depression/PTSD should consider improving social support and providing distress tolerance skills, in addition to housing.

## Figures and Tables

**Table 1:** Socio-demographic, behavioral, and health characteristics of the study sample of adults experiencing homelessness (n = 567).

Characteristics	N (%) or Mean (SD)
Mental health outcomes	
Depression	174 (30.7%)
PTSD symptoms	184 (32.5%)
Predictor variable	
Urban life stress	48.92 (14.86)
Mediator variables	
Distress tolerance	3.10 (0.98)
Perceived social support	32.95 (8.73)
Socio-demographic characteristics	
Age (years)	43.56 (12.0)
Sex	
Male	360 (63.5%)
Female	207 (36.5%)
Race/ethnicity	
White/non-minority	321 (56.6%)
Minority	246 (43.4%)
Marital status	
Married	70 (12.4%)
Not married	497 (87.6%)
Education	
Less than high school diploma	147 (25.9%)
High school diploma or higher	420 (74.1%)
Employment status	
Employed	66 (11.6%)
Unemployed/disability limiting employment	501 (88.4%)
Sources of income	
No source of income	305 (53.8%)
Have at least one source of income	262 (46.2%)
Health insurance	
No health insurance	399 (70.4%)
Insured to any extent	168 (29.6%)
Health behaviors	
Alcohol abuse/dependence	155 (27.3%)
Current smoker	444 (78.3%)
Homelessness characteristics	
Total amount of time homeless (years)	3.17 (4.31)
Shelter services utilization (number of services used)	4.48 (2.52)

Note: SD: Standard Deviation.

**Table 2:** Inter-correlations between participant characteristics (n = 567).

Variables	1	2	3	4	5
Main study variables					
Depression	1.00				
PTSD symptoms	0.41 ***	1.00			
Urban life stress	0.42 ***	0.38 ***	1.00		
Distress tolerance	−0.40 ***	−0.30 ***	−0.49 ***	1.00	
Perceived social support	−0.37 ***	−0.24 ***	−0.35 ***	0.36 ***	1.00
Covariates					
Age	0.05	0.04	0.02	0.02	−0.10 *
Sex (Female)	0.11 *	0.16 ***	0.10 *	−0.10 *	0.07
Race/ethnicity (White/non-minority)	0.09 *	0.01	0.01	−0.01	−0.05
Marital status (Married)	−0.04	−0.08	−0.001	−0.02	0.13 **
Education (Less than high school)	−0.01	−0.06	0.06	−0.12 **	−0.07
Employment (unemployed)	0.06	0.03	0.08 *	−0.02	−0.06
Income (no source of income)	0.04	−0.02	−0.01	−0.06	−0.01
Health insurance (uninsured)	−0.05	−0.06	0.02	−0.04	0.07
Alcohol abuse/dependence	0.06	0.13 **	0.17 ***	−0.12 **	−0.09 *
Current smoker	0.04	0.06	0.03	-0.06	−0.04
Total amount of time homeless	−0.02	0.06	0.02	0.001	−0.08
Shelter services utilization	0.01	0.04	0.02	−0.01	0.02

Note: Inter-correlations between study variables were evaluated using Pearson’s correlation for associations between continuous variables, point-biserial correlations for continuous-binary associations and Phi/Cramer V coefficients for associations between binary variables. * *p* < 0.5; ** *p* < 0.01; *** *p* < 0.001.

**Table 3:** Indirect effect of distress tolerance and perceived social support on the association between urban life stress and depression among homeless adults (n = 567).

Indirect Effect	b	SE	95% Bootstrap CI
Urban life stress → Distress tolerance → Depression	0.101	0.022	0.061, 0.147
Urban life stress → Perceived social support → Depression	0.091	0.020	0.053, 0.133
Urban life stress → Perceived social support → Distress tolerance → Depression	0.022	0.007	0.011, 0.037

Note: b = Standardized Coefficient, SE = Standard Error, CI = Confidence Interval.

**Table 4:** Indirect effect of distress tolerance and perceived social support on the association between urban life stress and PTSD among homeless adults (n = 567).

Indirect Effect	b	SE	95% Bootstrap CI
Urban life stress → Distress tolerance → PTSD symptoms	0.065	0.022	0.023, 0.112
Urban life stress → Perceived social support → PTSD symptoms	0.043	0.019	0.006, 0.082
Urban life stress → Perceived social support → Distress tolerance → PTSD symptoms	0.014	0.006	0.005, 0.026

Note: b = Standardized Coefficient, SE = Standard Error, CI = Confidence Interval, PTSD = Post-Traumatic Stress Disorder.
